# Replicates Number for Drug Stability Testing during Bioanalytical Method Validation—An Experimental and Retrospective Approach

**DOI:** 10.3390/molecules27020457

**Published:** 2022-01-11

**Authors:** Elżbieta Gniazdowska, Wojciech Goch, Joanna Giebułtowicz, Piotr J. Rudzki

**Affiliations:** 1 Łukasiewicz Research Network, Industrial Chemistry Institute, 8 Rydygiera, 01-793 Warsaw, Poland; elzbieta.gniazdowska@ichp.lukasiewicz.gov.pl or; 2 Department of Bioanalysis and Drugs Analysis, Doctoral School, Medical University of Warsaw, 61 Żwirki i Wigury, 02-091 Warsaw, Poland; 3 Department of Physical Chemistry, Faculty of Pharmacy, Medical University of Warsaw, 1 Banacha, 02-097 Warsaw, Poland; wojciech.goch@wum.edu.pl; 4 Department of Bioanalysis and Drugs Analysis, Faculty of Pharmacy, Medical University of Warsaw, 1 Banacha, 02-097 Warsaw, Poland; joanna.giebultowicz@wum.edu.pl; 5 Celon Pharma S.A., Bioanalytical Laboratory, 15 Marymoncka, 05-152 Kazuń Nowy, Poland

**Keywords:** confidence interval, stability, retrospective analysis, sample size, regulatory bioanalysis, bioanalytical method validation

## Abstract

Background: The stability of a drug or metabolites in biological matrices is an essential part of bioanalytical method validation, but the justification of its sample size (replicates number) is insufficient. The international guidelines differ in recommended sample size to study stability from no recommendation to at least three quality control samples. Testing of three samples may lead to results biased by a single outlier. We aimed to evaluate the optimal sample size for stability testing based on 90% confidence intervals. Methods: We conducted the experimental, retrospective (264 confidence intervals for the stability of nine drugs during regulatory bioanalytical method validation), and theoretical (mathematical) studies. We generated experimental stability data (40 confidence intervals) for two analytes—tramadol and its major metabolite (O-desmethyl-tramadol)—in two concentrations, two storage conditions, and in five sample sizes (*n* = 3, 4, 5, 6, or 8). Results: The 90% confidence intervals were wider for low than for high concentrations in 18 out of 20 cases. For *n* = 5 each stability test passed, and the width of the confidence intervals was below 20%. The results of the retrospective study and the theoretical analysis supported the experimental observations that five or six repetitions ensure that confidence intervals fall within 85–115% acceptance criteria. Conclusions: Five repetitions are optimal for the assessment of analyte stability. We hope to initiate discussion and stimulate further research on the sample size for stability testing.

## 1. Introduction

Evaluation of drug or metabolite stability in biological samples in conditions reflecting sample handling and analysis during bioanalytical method validation is recommended by international regulatory guidelines [1,2] and ICH M10 draft guidelines [3]. This evaluation includes stability in the biological matrix (short-term, long-term, and freeze-thaw), in processed samples and solutions (stock and working solutions). Kaza et al. (2019) [4] discussed the differences and similarities in bioanalytical method validation guidelines [1,2], but the authors omitted to mention differences in the recommended sample size (number of samples) for stability testing. The European Medicines Agency (EMA) [1] does not recommend any specific sample size whereas the U.S. Food and Drug Administration (FDA) [2] and ICH [3] recommend a minimum of three quality control samples (QC) per level of concentration of low QC and high QC to assess the stability of an analyte in a biological matrix. A note from Health Canada does not recommend examining stability using only one repetition of a QC sample [5].

The analyte stability testing refers to other characteristics of the bioanalytical method. The calibration range helps to select studied concentrations (low- and high-quality control samples). However, method precision is important to compare reference samples (e.g., prepared ex tempore) and test samples (i.e., stored for a specified time in specified conditions). Before any regulatory bioanalytical method validation guideline was published, Timm et al. proposed a stability assessment incorporating the precision in the calculation of 95% confidence intervals [6]. However, its application was limited by the assumed equality of variances for the reference and test samples. Rudzki and Leś extended this method for datasets with unequal variances [7]. They also proposed the use of 90% confidence intervals instead of 95% [6] to make the probability equal to the bioequivalence recommendations [8]. Confidence intervals are a good tool for testing stability. Since their introduction by Jerzy Spława Neyman in 1936 [9] they became widely used, including clinical research—for example as bioequivalence criterium [8]. Briefly, the idea of confidence intervals is to define a range of values describing parameters of interest in the population, based on parameter estimates observed in the sample. This estimation has a defined probability—usually 90%, 95%, or 99%. For example, a 90% confidence interval of 85.1–105.2% for mean stability means that there is a 90% probability that the mean stability is between 85.1% and 105.2%. In the case of stability testing, the confidence interval combines central tendency (mean difference between stored and reference samples) and data dispersion (method precision) with a selected probability. This approach is not yet frequently used because it is more restrictive and labor intensive than the guidelines’ recommendations. Nevertheless, the confirmation of analyte stability in a biological matrix using this method is associated with a low and pre-defined probability of true instability.

The stability assessment proposed in the draft of the ICH M10 bioanalytical method validation guideline [3] recommends analyzing stored and reference samples but does not include a description of any comparison between them. The lack thereof creates the risk of accepting the method regardless of the 29.8% instability of an analyte [4]. Moreover, there is an insufficient justification of sample size (number of samples) in the stability evaluation. Limiting testing to three samples in each dataset may lead to stability results biased by a single outlier. However, how much do additional analyses increase confidence in the stability results? Is this increase relevant? How to balance it with the cost of extra analyses? Although there may be no universal answer to these questions, further research on sample size for stability assessment is needed.

In this paper, we aim to evaluate the optimal sample size for drug stability testing in human plasma based on confidence intervals [6,7] by conducting an experimental study for tramadol and its major metabolite (O-desmethyl-tramadol), as well as a retrospective data analysis for nine drugs of different structure. 

## 2. Materials and Methods

### 2.1. Materials

O-desmethyl-tramadol hydrochloride (≤99%) was purchased from LoGiCal (Luckenwalde, Germany) and tramadol hydrochloride (≤99%) was purchased from Saneca Pharmaceuticals (Hlohovec, Slovakia). O-desmethyl-tramadol-d6 (≤98%) and tramadol-d6 hydrochloride (≤99%) were purchased from TLC Pharmaceutical Standards (Newmarket, Ontario, Canada). All other reagents were of analytical grade. Methanol and formic acid were purchased from Merck KGaA (Darmstadt, Germany). Sodium hydroxide was obtained from Chempur (Piekary Śląskie, Poland). Human blank plasma with CPD (citrate, phosphate, dextrose) as an anticoagulant was obtained from the Regional Blood Donation and Blood Therapy Centre (Warsaw, Poland).

### 2.2. Mass Spectrometric and Chromatographic Conditions

The bioanalytical method was adapted from the previous study [10] with a different chromatographic column and the use of formic acid in the mobile phase instead of acetic acid. The adapted method was validated according to the EMA [1] guidelines, except for long-term stability which was confirmed previously. Instrumental analysis was performed on an Agilent 1260 Infinity (Agilent Technologies, Santa Clara, CA, USA), equipped with an autosampler, a degasser, and a binary pump coupled to a hybrid triple quadrupole/linear ion trap mass spectrometer QTRAP 4000 (ABSciex, Framingham, MA, USA). The Turbo Ion Spray source was operated in positive mode with voltage and source temperatures of 5500 V and 550 °C, respectively. The curtain gas, ion source gas 1, ion source gas 2, and collision gas (all high purity nitrogen) were set at 206.84 kPa, 275.79 kPa, 379 kPa, and “high” instrument units, respectively. The target compounds were analyzed in the Multiple Reaction Monitoring (MRM) mode (Table 1).

Chromatographic separation was achieved with a Kinetex C18 column (100 mm × 4.6 mm, 2.6 μm, Phenomenex, Torrance, CA, USA) using isocratic elution with methanol and 0.1% formic acid in a ratio of 40:60 at a flow rate of 0.3 mL/min. The column and the autosampler temperature was 50 ± 1 °C and 20 ± 1 °C, respectively. The injection volume was 5 μL.

### 2.3. Stock Solution, Calibration Standards, and Quality Control Samples

The separate standard stock solutions of tramadol, O-desmethyl-tramadol, tramadol-d6, and O-desmethyl-tramadol-d6 were prepared in 50% methanol (*v/v*) and were stored at −20 °C. The standard working solution was prepared by mixing stock solutions with an appropriate volume of water. The internal standard working solution (250 ng/mL for tramadol-d6 and 75 ng/mL for O-desmethyl-tramadol-d6) was diluted with water and prepared by mixing both internal standards stock solutions.

All calibration standards and the quality control samples were prepared by spiking blank human plasma with a working solution containing both analytes. The calibration standards contained both tramadol and O-desmethyl-tramadol at eight concentrations ranging from 5.0 to 750 ng/mL and from 2.5 to 150 ng/mL. The quality control samples were prepared at concentrations of 15, 350, and 600 ng/mL for tramadol, and 7.5, 70, and 120 ng/mL for O-desmethyl-tramadol. 

### 2.4. Sample Preparation

The liquid-liquid extraction with *tert*-butyl methyl ether and 1M sodium hydroxide was used for the sample preparation [10]. Internal standards were added in one solution. The ether phase was evaporated in nitrogen gas and the dry residue was reconstituted with 150 μL of the mobile phase.

### 2.5. Stability Evaluation and Statistical Methods

The short-term stability was evaluated with sets containing an equal number of test and reference-quality control samples (QC): 3, 4, 5, 6, and 8 for low QC (15/7.5 ng/mL tramadol and O-desmethyl-tramadol) and high QC (600/120 ng/mL tramadol and O-desmethyl-tramadol). The reference and test QC samples (plasma fortified with tramadol and O-desmethyl tramadol solution) were prepared. The test QC samples were stored at room temperature for 24 and 72 h before extraction and LC-MS analysis. Autosampler stability test during the validation method, confirmed that samples are stable for a minimum of 68 h at room temperature [10]. Reference samples were analyzed immediately after preparation, after 24 and 72 h storage in an autosampler at 20 ± 1 °C in the same sequence as test samples. Acceptance criteria were met when the whole confidence interval was within the acceptance range of 85–115%. 

The statistical analysis of stability was based on the application of 90% confidence intervals [6,7]. The F-Snedecor test (significance level α = 0.01) was applied to test the hypothesis on variance equality. The influence of the number of repetitions and analyte concentration on the position and width of the confidence interval was analyzed using an analysis of variance (ANOVA, *p* = 0.05) test with repeated measurements. Normal distribution of the stability was assumed in the estimation of the probability that the confidence interval width is below 30%. The probability P(CI⊂[85; 115]) was calculated using the equation:

$$P\left(CI\subset \left[85;\;115\right]\right)=\chi_{n-1}\left(\frac{225\;n\;\left(n-1\right)}{k^{2}\;\sigma_{S}{}^{2}}\right)$$

where:

χ*_n_*_−1_—cumulative distribution function of the chi-square distribution for degrees of freedom (df) = *n* − 1;
*n*—number of repetitions;
*k*—the value of the Student t-distribution quantile at a 0.1 significance level for *n* − 1 degrees of freedom (df);
$\sigma_{S}$—standard deviation in stability.

More details on mathematical calculations can be found in the Appendix A. 

### 2.6. Retrospective Analysis

Stability results for nine drugs were recorded during method validations conducted under Good Laboratory Practice conditions at the former Pharmaceutical Research Institute in Warsaw, Poland ([11,12,13,14,15], and unpublished data). The following types of stability were studied: short-term stability, freeze and thaw stability, long-term stability at temperatures of −14 °C and −65 °C. Nine drugs with LC-MS and HPLC-UV methods of determination of varying precision were selected to create the data sets. For each drug and each stability test, *n* = 6 samples were recorded at each low and high QC concentration. To analyze the worst-case scenario, for each dataset a result lying nearest to the mean of *n* = 6 results was discarded to obtain *n* = 5 dataset. The same procedure was used to obtain datasets of *n* = 4 and *n* = 3. The final number of calculated confidence intervals was 264. Comparison of the width of the confidence intervals between low and high QC was made using a Wilcoxon signed-rank test (significance level *p* < 0.05). To analyze how differences in one variable (percentage of confidence intervals within acceptance criteria set at 85–115%) can be explained by a difference in a second variable (confidence width or the number of samples), the coefficient of determination was used.

## 3. Results

### 3.1. Experimental and Mathematical Studies

**Figure 1:**
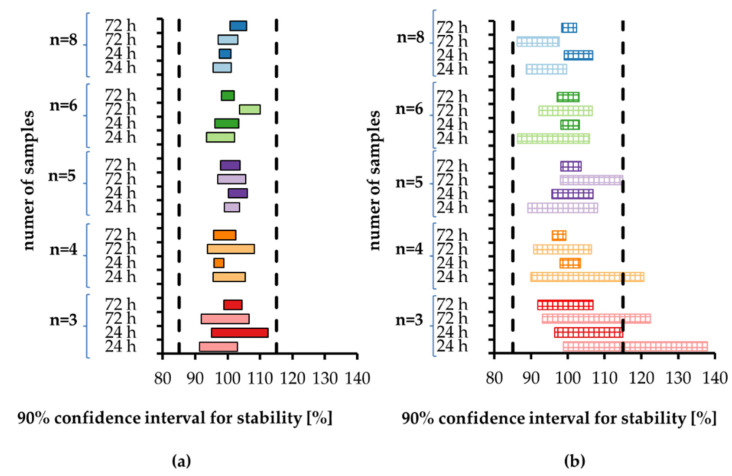
The 90% confidence intervals for the stability calculated according to [7] for (**a**) tramadol and (**b**) O-desmethyl-tramadol in human plasma stored at room temperature for 24 h and 72 h. Each sample size is associated with a different color, with light color indicating low concentration and dark color indicating high concentration. Vertical dashed lines indicate stability limits of 85–115%.

Thanks to the design of the experimental study (five sample sizes, two storage durations, two analytes in two concentrations each) we were able to calculate 40 confidence intervals (Figure 1). For 20 pairs of low and high QC concentrations, we recorded 18 cases (90%) where the 90% confidence interval was wider for low than for high concentration. Moreover, the variability of the confidence interval width—presented as relative standard deviation (RSD) in Table 2—was larger for low concentration. It shows the influence of method precision on stability evaluation, as lower concentrations were measured with worse precision. 

Moreover, wider confidence intervals for low concentrations of O-desmethyltramadol than for low concentrations of O-tramadol indicate the importance of method precision. The precision of O-desmethyltramadol determination in quality control samples was 7.38% for low QC (7.5 ng/mL) and 2.90% for high QC (120 ng/mL). The precision of tramadol determination was 6.43% for low QC (15 ng/mL) and 3.07% for high QC (600 ng/mL). For each studied QC level, the mean extraction recovery was consistent for both analytes and their ISs—86.08–87.99% for tramadol, 85.55–86.99% for tramadol-d6, 74.45–78.75% for O-desmethyltramadol, and 74.61–79.07% for O-desmethyltramadol-d6. Thus, we do not expect that extraction recovery influenced stability results.

**Figure 2:**
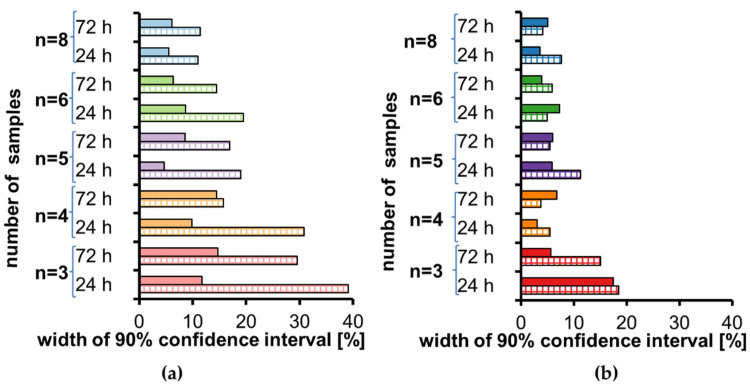
Width of a 90% confidence interval for stability calculated according to [7] for tramadol (full color) and O-desmethyl-tramadol (striped color) in human plasma stored at room temperature for 24 h and 72 h for each sample size: (**a**) low concentration, (**b**) high concentration.

Visual assessment of low concentration data (Figure 2a) indicates that three and four repetitions are not appropriate due to the width of some confidence intervals over 30%. For five and six repetitions, width is below 20%, while for eight repetitions, width is below 12%. Visual assessment of high concentration data (Figure 2b) is a bit different. For three repetitions the confidence intervals width in 3/4 cases is over 15%, while for all other repetitions it is below 8%, with one exception of 11% (*n* = 5).

**Figure 3:**
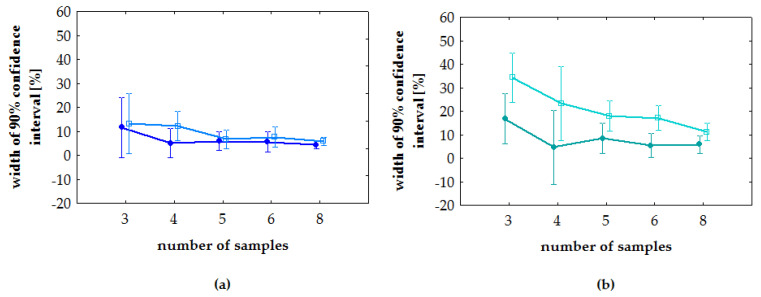
Post-hoc least significant difference test (Fisher’s LSD test). Vertical bars means a 90% confidence interval: (**a**) tramadol; (**b**) O-desmethyl-tramadol. On each plot, light color indicates low concentration and dark color indicates high concentration.

ANOVA showed no dependence of the width of the confidence interval on the analyte concentration (Figure 3) (*p* > 0.1187). Results of the post-hoc least significant difference test (Fisher’s LSD test) for sample size showed that the width of the confidence interval for *n* = 3 statistically significantly differs from more repetitions (*n* = 4, 5, 6, 8) (*p* from <0.0001 to 0.0249). The width of the confidence interval for *n* = 4 differs only from eight repetitions (*p* < 0.05). 

**Figure 4:**
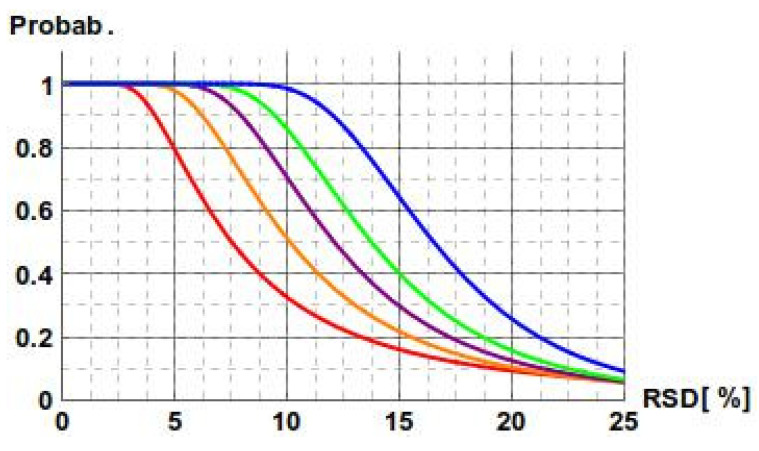
Dependence of the probability that the confidence interval width is below 30% on the precision in measurements. Equal precision for the reference and the studied measurements is assumed. Curves are defined for *n* = 3 (red), *n* = 4 (orange), *n* = 5 (purple), *n* = 6 (green), and *n* = 8 (blue).

Additionally, we have investigated the relation between precision, confidence interval, and the number of repetitions. The length of the confidence interval depends on the sample variance—the greater the *n*, the shorter the length of the interval (as it is inversely proportional to the square root of *n*), and the higher the chance the sample variance is assessed correctly. We calculated the probability that for a given precision, the confidence interval derived from *n* repetitions falls within a 30% range. As expected, the relation between precision and the number of repetitions is sharp (Figure 4). As an example, for 10% precision, the considered probability is 33% for *n* = 3, 51% for *n* = 4, 71% for *n* = 5, 86% for *n* = 6, and 98% for *n* = 8. In general, for a smaller number of repetitions, there is a significant probability that the measurements with even high precision may overestimate the sample variance and consequently the length of the confidence interval. The choice of five or six repetitions proves to be enough to ensure that the confidence intervals will fall within the 85–115% interval. 

We postulate that five repetitions of quality control samples at low and high concentration levels are optimal for stability tests during bioanalytical method validation. For each case with *n* = 5, the stability tests passed and the width of all confidence intervals was below 20%. For *n* < 5 some of the stability tests failed (part of the confidence interval outside of the acceptance criteria of 85–115%) due to the width of confidence intervals exceeding 30%. Moreover, for *n* > 5 all stability tests passed and the mean width of the confidence intervals decreased gradually (Table 2). 

### 3.2. Retrospective Study

**Figure 5:**
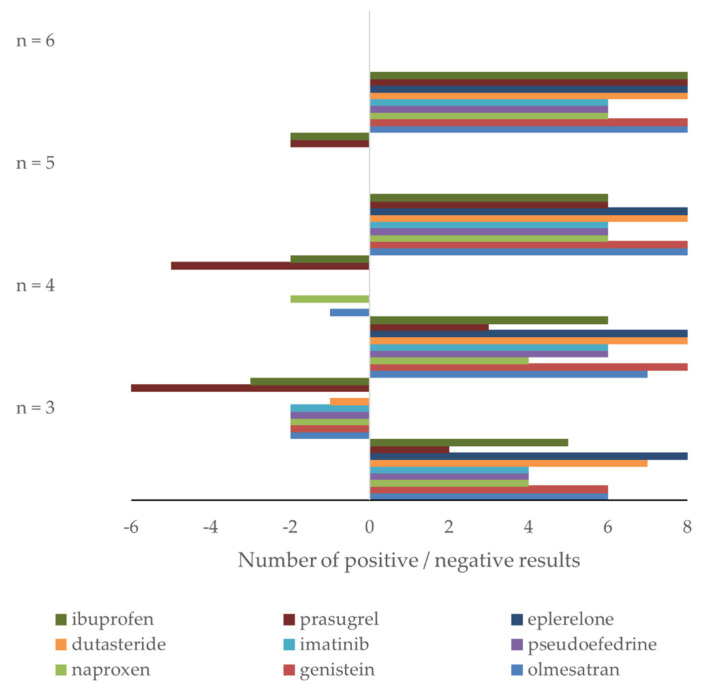
Retrospective study of nine drugs’ stability in human plasma: number of confidence intervals within (positive results) and outside (negative results) acceptance criteria for nine drugs using *n* = 3, 4, 5, and 6 samples for stability testing. High and low concentration data are combined.

**Figure 6:**
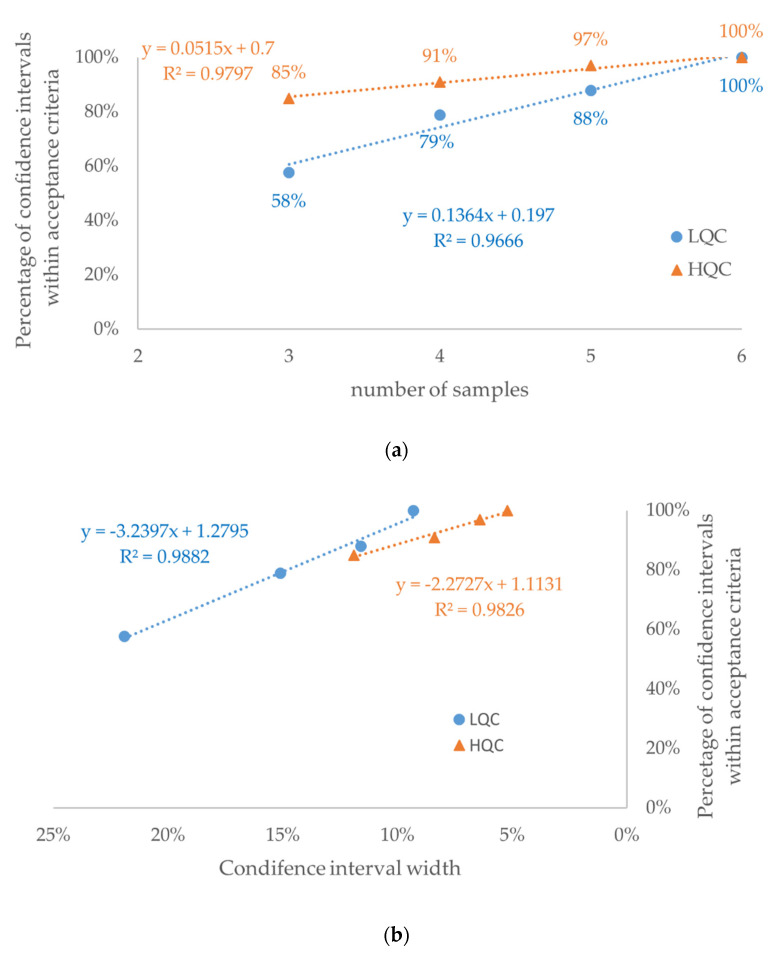
Retrospective study of nine drugs’ stability in human plasma: percentage of confidence intervals within acceptance criteria in the function of (**a**) number of samples and (**b**) mean width of the confidence interval for each number of samples (see Table 2). The dataset consisted of 33 confidence intervals for each concentration level: LQC (circle)—low-quality control sample; HQC (triangle)—high-quality control sample.

**Figure A1:**
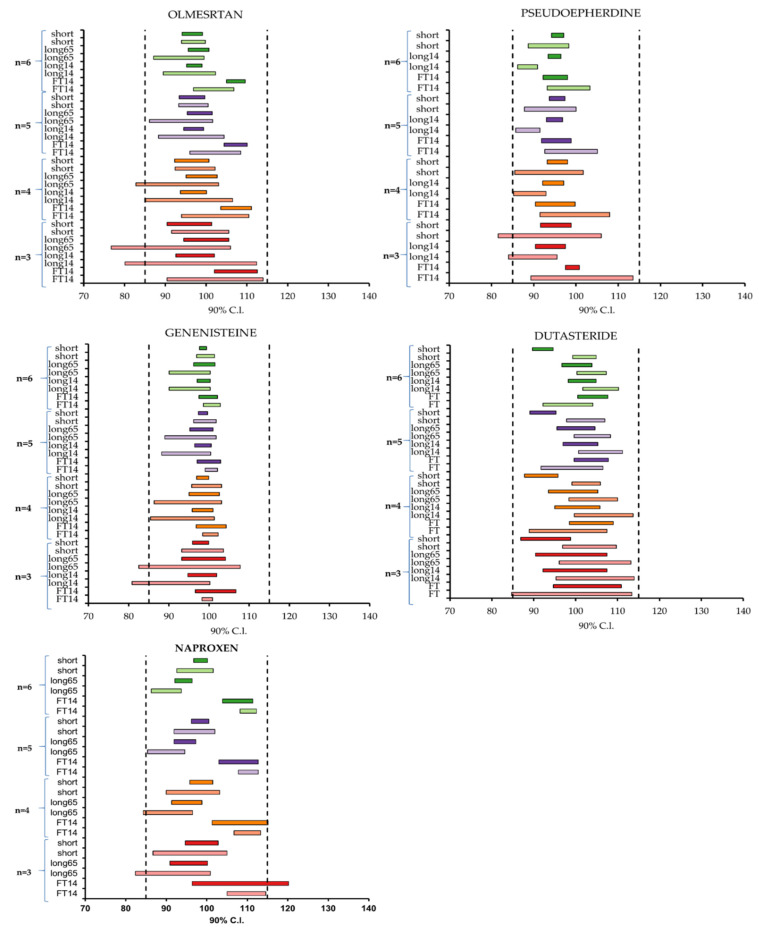

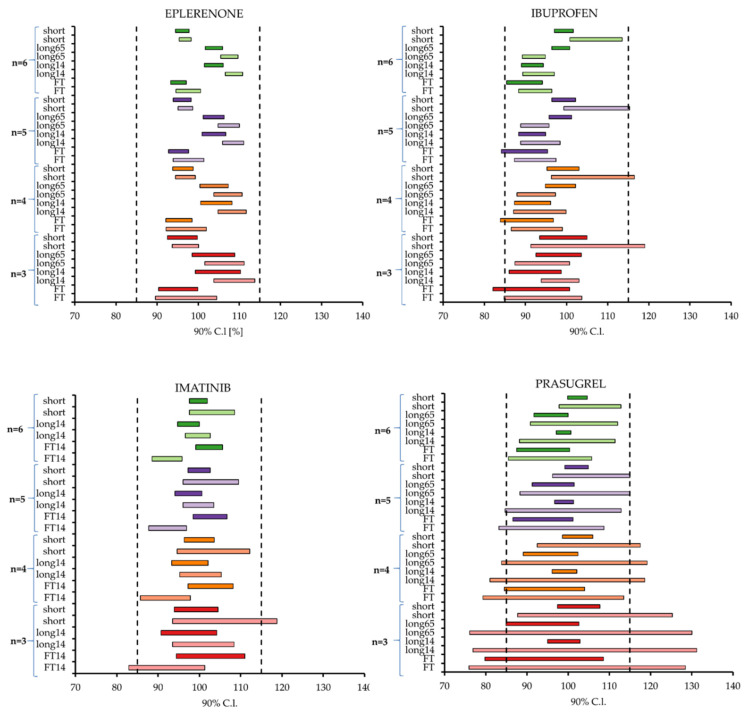
Retrospective study: 90% confidence intervals (90% C.I) for the stability of five drugs using *n* = 3, 4, 5, and 6. Abbreviations: Short—short-term stability, FT—freeze and thaw stability, long—long-term stability. Numbers 14 and 65 indicate the storage temperature of −14 °C and −65 °C, respectively. Low QC—light color, high QC—intensive color.

To verify observations from the experimental and the theoretical studies, we have analyzed human plasma stability data for nine validated bioanalytical methods (Figure 5 and Figure A1). For all data, the percentage of confidence intervals lying within acceptance criteria was acceptable for *n* = 5 (88% for low and 93% for high concentration, respectively) and reached 100% for *n* = 6 (Figure 6a). For *n* = 5, only 5 of 66 results (including four for low QC) were outside of the acceptance limits. The greatest difference between the confidence interval limits and the acceptance criteria was 1.8%.

**Figure 7:**
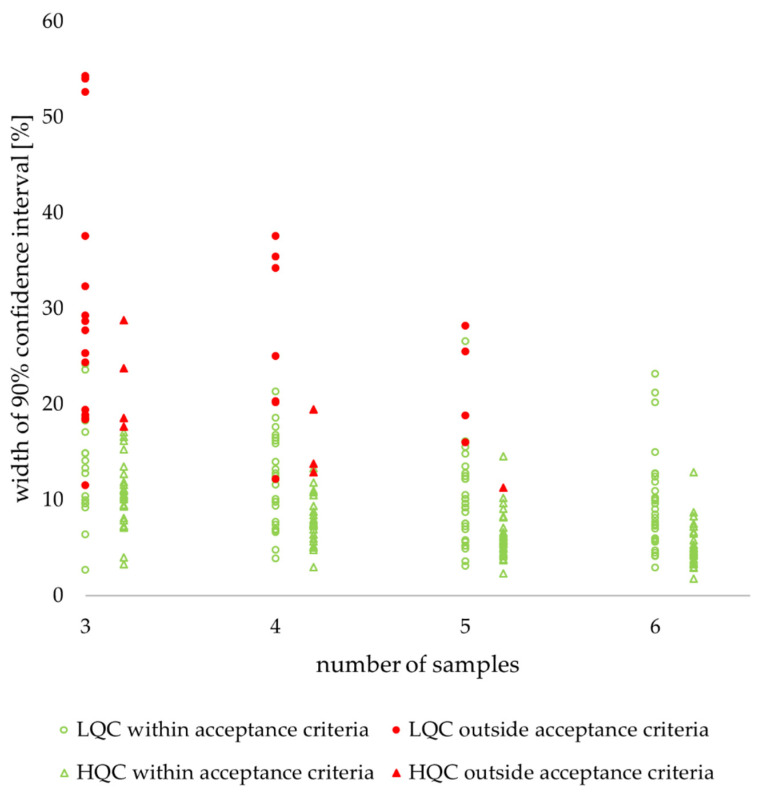
Retrospective study of nine drugs’ stability in human plasma: individual values of confidence interval width. LQC (circle)—low-quality control sample, HQC (triangle)—high-quality control sample. Filled red figures indicate values outside acceptance criteria, unfilled green figures indicate values within acceptance criteria.

**Figure A2:**
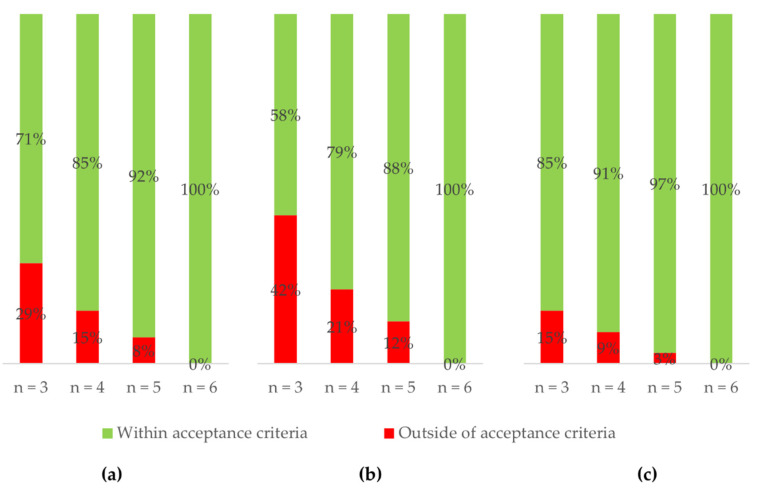
Retrospective study: Percentage of confidence intervals outside (red) and within (green) stability acceptance criteria for (**a**) all data, (**b**) low QC concertation, and (**c**) high QC concertation. Combined data from a retrospective study of nine drugs using *n* = 3, 4, 5, and 6.

As expected, a strong positive correlation (r^2^ > 0.96) was observed between the number of samples and the percentage of confidence intervals within the acceptance criteria (Figure 6a). Consequently, a strong negative correlation (r^2^ > 0.98) was observed between the confidence interval width and the percentage of confidence intervals within the acceptance criteria (Figure 6b). Among confidence intervals for *n* = 3, 4, and 5, more than a 2-fold higher percentage of confidence intervals outside of acceptance criteria was observed for the low QC (Figure A2b) than for the high QC (Figure A2c) concentration (*p* < 0.00001). This observation is consistent with higher values of both width of the confidence interval and its variability expressed as RSD (Table 2, Figure 7 and Figure A4). 

**Figure A3:**
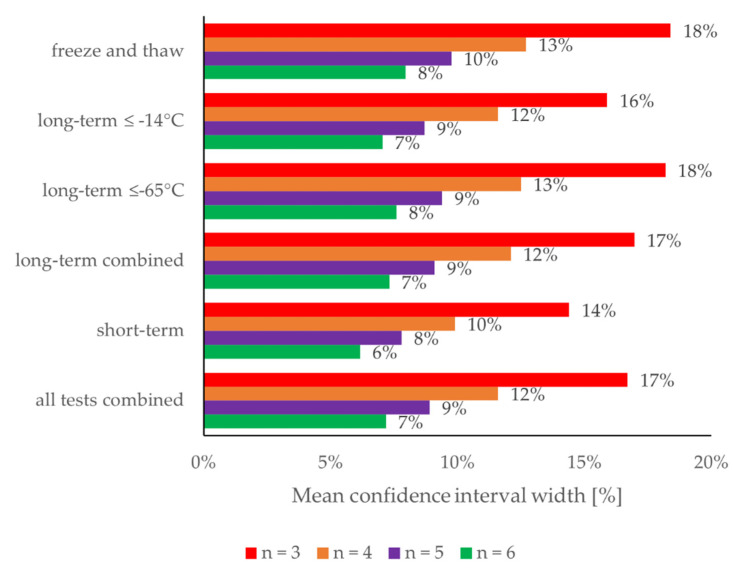
Retrospective study: Relation between mean confidence interval width and type of stability test.

**Figure A4:**
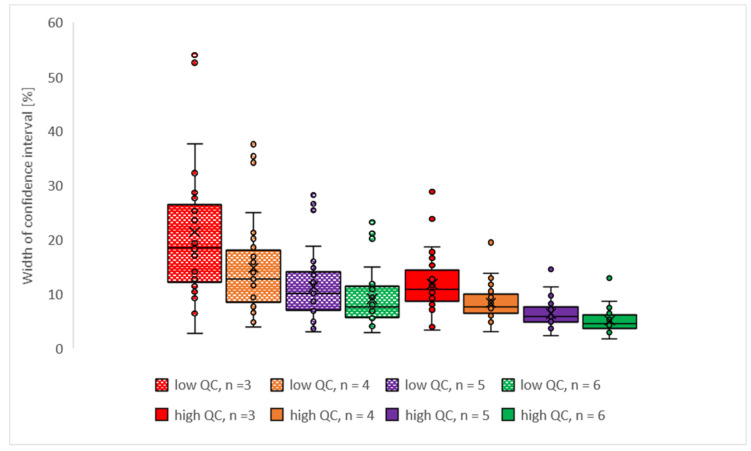
Retrospective study: box & whiskers plot; box indicates 2nd and 3rd quartile, whiskers indicate 1st and 4th quartile, points outside of whiskers indicate outliers.

There were no relevant differences in confidence interval width between stability tests (Figure A3). The highest values for all sample numbers were recorded for the freeze and thaw test, but all other values for each sample number were only 1–2% lower.

## 4. Discussion

The results of the experimental, theoretical, and retrospective studies are in good agreement indicating that using 90% confidence intervals requires testing of at least five repetitions of quality controls as references and as stability samples. A retrospective study revealed that the percentage of the confidence intervals within acceptance criteria is strongly correlated with the number of samples used for stability testing (positively) and the mean of the width of confidence intervals (negatively). The statistically significant difference between low QC and high QC was observed between the percentage of confidence intervals within the acceptance criteria for a given sample number. The type of stability test did not influence confidence interval width. It seems that the excess work between *n* = 5 and *n* = 8 is not balanced with the benefit of a narrower width of the confidence interval. On the other hand, there would be 72 more analyses during full validation for one analyte, and this number does not include stability testing in solutions. The amount of excess work and resources for additional analyses may not be assessed in general, because it depends on particular method characteristics.

Our experimental study used a single bioanalytical method for the determination of two analytes in a single laboratory. To increase confidence in conclusions, we have reused previously generated stability data for nine drugs. Retrospective analyses are very popular in medicine [16,17], and slightly less popular in pharmacy [18,19]. On the contrary, in analytical chemistry retrospective analyses are used very rarely [20]. Over 20 years ago the concept of green analytical chemistry to protect the environment was established. Recently, its extension was proposed: white analytical chemistry in addition to green aspects also takes into account analytical and practical attributes [21]. Nevertheless, retrospective analysis has even greater ecological aspects since no chemical analysis is required and no waste is generated. Considering the high amount of analytical data produced each year in laboratories, it would be beneficial to explore them all deeply to draw some general conclusions, answer the emerging questions, and contribute to international guidelines development. The retrospective study enabled comparison of data generated using LC-MS and HPLC-UV methods (Table A3). It may be observed that narrower stability confidence intervals were recorded for HPLC-UV determined imatinib than for LC-MS/MS determined prasugrel (Figure A1). On the other hand, narrower stability confidence intervals were recorded for LC-MS determined eplerenone than for HPLC-UV determined ibuprofen (Figure A1). This indicates that the detector type and concentration range are not the appropriate indicator of confidence interval width, which is dependent on method precision.

We limited our study to plasma samples. For neat solutions, due to the lower probability of interferences and lack of variability introduced by sample preparation, the precision should be better and the optimal number of repetitions could be lower. We avoided the exclusion of outlying results. An alternative approach is to use a smaller number of replicates and remove outliers using statistical tests such as the Q-Dixon or Grubbs test. However, this approach—especially for a small number of replicates—may provoke questions from regulatory agencies. Additionally, it does not take into account the precision of the method. Therefore, we do not recommend this approach. The limitation of the retrospective study is that all confidence intervals for *n* = 6 were within the acceptance criteria as we used validated methods. The calculation of a 90% confidence interval may be considered as complicated compared to current bioanalytical method validation guidelines [1,2]. However, an extra effort in data analysis increases the reliability of stability evaluation.

We assumed a normal distribution of concentration data for stability and reference samples. However, stability is a ratio of stability samples over reference samples and the ratio of two normally distributed samples is never normally distributed itself. This statistical issue is taken into account for bioequivalence testing where the acceptance criteria of 80–125% does not center symmetrically around 100% but does so in log space. Thus, acceptance limits of 85–115% may not be appropriate for stability testing. An approach similar to bioequivalence suggests a criterion of 85.00–117.65%. We have opted to use 85–115% acceptance limits, which are well-established in regulatory guidelines [1,2], but their inconsistency with stability distribution needs further consideration. 

Our results are important because the current recommendation of at least three samples for stability testing [2,3] is not sufficient. The proposed *n* = 5 is in line with reports from other laboratories [22,23,24] where five or six results were used to calculate the 90% confidence intervals for stability. Extending stability acceptance criteria from deviation from nominal concentration by adding a test-to-reference ratio may be considered as an increase of regulatory burden. On the other hand, the reliability of bioanalytical data is crucial for pharmacokinetic calculations and decisions on dosing schemes. The latter impacts drug efficiency and patient safety. Thus, the proper balance between too extensive testing and poor data quality requires further discussion. A possible answer may be a hybrid approach: hard criteria for deviation of the mean from nominal concentration combined with soft criteria for the 90% confidence interval for test-to-reference ratio.

Both experimental and retrospective studies suggest that an optimal number of repetitions is five, as also recommended by the European Bioanalysis Forum [25]. The proper assumption on the relationship between method precision and sample size may be a key factor for successful future simulations. We hope that this paper will initiate discussion and stimulate further research on optimal sample size for stability testing. We expect that further simulations and retrospective studies from other laboratories will support the need for bioanalytical guidelines update.

## 5. Conclusions

Five sample repetitions are optimal for the assessment of analyte stability during bioanalytical method validation. Experimental, theoretical, and retrospective study results led to similar conclusions. The number of three or four replicates, in spite of being acceptable in some guidelines, is insufficient (in some cases, the width of the confidence intervals for stability exceeded 30%, which precluded meeting the acceptance criteria). In contrast, the excess work between *n* = 5 and *n* = 8 was not balanced with any benefit of narrower confidence interval widths. We hope to initiate a discussion on sample size for stability studies. Such a discussion may result in updated bioanalytical method validation guidelines.

## Figures and Tables

**Table 1:** Parameters of MS method.

	Retention Time (min)	MRM [*m/z*]	DP [V]	CE [V]	CXP [V]
tramadol	3.4	264.2 > 42.3	51	125	10
tramadol-d6	3.4	270.3 > 252.2	66	17	16
O-desmethyl-tramadol	2.6	250.2 > 232.2	71	17	18
O-desmethyl-tramadol-d6	2.6	256.0 > 238.3	61	17	14

MRM—multiple reaction monitoring; DP—declustering potential; CE—collision energy; CXP—cell exit potential.

**Table 2:** Descriptive statistics for the width [%] of a 90% confidence interval. The number of pairs is the equal number of reference and study samples.

	Low QC					High QC				
Number of Pairs	3	4	5	6	8	3	4	5	6	8
**Experimental Data for Tramadol and O-desmethyl-tramadol (*n* = 4 of results at each column)**										
Mean	23.8	17.7	12.4	10.2	8.5	14.2	4.8	7.2	5.5	5.1
Geometric mean	21.1	16.2	10.7	9.7	8.1	12.9	4.6	6.9	5.4	4.9
Median	22.1	15.1	12.8	9.9	8.6	16.3	4.7	6.0	5.5	4.6
Min	11.7	9.9	4.7	6.4	5.5	5.7	3.1	5.5	3.9	3.6
Max	39.1	30.8	19.5	14.5	11.4	18.5	6.8	11.4	7.3	7.7
SD	12.9	9.1	6.9	3.5	3.1	5.8	1.7	2.8	1.4	1.8
RSD [%]	54	51	56	34	37	41	35	39	26	36
**Retrospective Analysis (*n* = 33 of results at each column)**										
Mean	21.5	14.9	11.4	9.1	-	11.9	8.4	6.4	5.1	-
Geometric mean	18.0	12.9	9.9	8.1	-	10.8	7.9	6.0	4.8	-
Median	18.5	12.8	10.1	7.7	-	10.9	7.6	5.8	4.6	-
Min	2.7	3.9	3.1	2.9	-	3.3	3.0	2.3	1.8	-
Max	54.3	37.6	28.2	23.2	-	28.8	19.5	14.6	12.9	-
SD	13.0	8.4	6.3	5.0	-	5.2	3.2	2.5	2.1	-
RSD [%]	57	54	52	53	-	43	38	38	40	-

## Data Availability

Data presented in Appendix A.

## Appendix A

**Table A1:** Concentrations of tramadol in human plasma during stability testing after storage for 24 h and 72 h at room temperature—low QC (nominal concentration of 15.0 ng/mL) and high QC (nominal concentration of 600 ng/mL).

Number of Samples (n)	Low QC (ng/mL)				High QC (ng/mL)			
	Reference for 24 h	Tested 24 h	Reference for 72 h	Tested 72 h	Reference for 24 h	Tested 24 h	Reference for 72 h	Tested 72 h
8	14.9	14.5	14.2	14.6	573	567	590	640
	15.2	14.7	14.6	14.6	582	569	594	627
	15.4	14.8	14.7	14.6	584	574	572	611
	15.5	15.4	14.9	14.9	586	575	589	578
	15.6	15.4	15.1	14.9	588	579	580	617
	15.8	15.5	15.3	14.9	590	587	609	609
	15.9	15.6	15.5	15.4	594	591	586	584
	16.0	16.3	15.7	16.2	597	615	582	589
6	14.7	14.0	13.9	14.4	568	559	574	582
	14.8	14.5	13.9	15.1	573	564	578	585
	15.1	14.9	14.4	15.3	577	567	583	587
	15.2	15.1	14.5	15.6	587	583	596	591
	15.7	15.3	14.5	15.7	598	591	603	594
	16.3	16.0	14.7	15.7	600	630	606	602
5	14.7	14.8	14.9	14.5	555	572	553	569
	14.9	14.9	15.0	14.8	559	579	585	581
	14.9	15.2	15.1	15.2	566	584	589	588
	15.0	15.4	15.2	15.8	578	601	590	596
	15.4	15.6	15.2	16.1	591	601	594	599
4	14.3	14.3	13.8	14.3	594	571	579	559
	14.9	14.3	14.9	14.7	594	582	579	567
	14.9	15.1	15.5	15.5	595	585	582	592
	15.0	15.7	15.7	15.8	606	588	597	597
3	15.0	14.9	14.6	14.7	570	565	575	584
	15.6	15.2	15.4	15.4	574	578	589	598
	16.4	15.5	16.2	15.6	580	642	592	603

**Table A2:** Concentrations of O-desmethyl tramadol in human plasma during stability testing after storage for 24 h and 72 h at room temperature—low QC (nominal concentration of 7.50 ng/mL) and high QC (nominal concentration of 120 ng/mL).

Number of Samples (n)	Low QC (ng/mL)				High QC (ng/mL)			
	Reference for 24 h	Tested 24 h	Reference for 72 h	Tested 72 h	Reference for 24 h	Tested 24 h	Reference for 72 h	Tested 72 h
8	7.59	6.87	6.74	6.40	112	132	117	118
	7.21	6.88	7.07	6.47	111	115	118	119
	7.65	6.90	7.55	6.66	112	117	118	121
	7.89	7.22	7.64	6.70	114	117	122	122
	7.93	7.41	7.79	6.95	117	117	122	122
	8.16	7.67	7.92	7.34	117	118	123	123
	8.36	8.02	7.94	7.68	119	119	123	124
	8.37	8.52	8.28	7.69	126	119	127	124
6	7.89	6.37	7.16	6.61	114	117	117	125
	7.35	6.44	7.21	6.81	116	118	118	116
	7.77	8.08	7.23	7.11	116	118	119	119
	8.11	8.19	7.46	7.45	120	120	124	122
	8.22	8.31	7.73	8.04	120	120	124	122
	8.25	8.36	7.82	8.32	124	121	125	123
5	7.02	7.85	6.64	6.87	119	124	117	119
	7.48	6.24	6.71	7.16	113	111	120	120
	7.52	7.70	6.72	7.33	113	114	121	121
	7.87	7.84	7.37	7.69	117	115	122	122
	8.20	7.84	7.68	8.22	122	127	124	127
4	6.98	7.66	6.94	6.40	117	116	122	118
	6.42	7.52	7.02	6.83	117	118	122	119
	8.39	7.55	7.09	6.97	119	119	124	120
	8.46	8.61	7.33	7.74	120	123	124	123
3	7.05	7.64	6.70	6.41	123	117	115	120
	6.39	8.54	6.84	7.54	116	125	127	122
	8.01	8.80	6.88	7.93	117	133	128	124

**Table A3:** Characteristics of the bioanalytical methods for the determination of the nine drugs used for retrospective analysis.

Drug	Method	Internal Standard	Low/High QC (ng/mL)	Type of Extraction	Source
Dutasteride	HPLC, ESI +	[^13^C_6_]-dutasteride	0.3/2.8	LLE	[24]
Eplerenon	HPLC-MS, ESI +	[^2^H_3_]-eplerenone	50/1500	LLE	[23]
Genistein	HPLC-MS, ESI −	[^2^H_4_]-genistein	50/2000	LLE	N/A
Ibuprofen	HPLC-UV, λ = 220 nm	naproxen	900/24,000	LLE	N/A
Imatinib	HPLC-UV, λ = 265 nm	propranolol hydrochloride	120/3200	LLE	[22]
Naproxen	HPLC-UV, λ = 265 nm	ibuprofen	1500/60,000	LLE	[20]
Olmesartan	HPLC-MS, ESI +	[^2^H_6_]-olmesarta	15/2000	LLE	[21]
Prasugrel	HPLC-MS/MS, ESI +	[^13^C_6_] R-138727	1.5/200	LLE	N/A
Pseudoephedrine	HPLC-MS/MS, ESI +	[^2^H_3_][^13^C_6_]-pseudoephedrine	4.5/240	LLE	N/A

LLE—liquid-liquid extraction; ESI—electrospray ionization; N/A—unpublished data.

**Relation between precision in measurements and stability:**

Stability S is determined as a ratio of two uncorrelated random variables *X* (tested samples) and *Z* (reference samples):

$$S=\frac{X}{Z}$$

Our goal is to derive the relation between the standard deviations in *X* and *Z* and the standard deviation in *S*. We start with linearization, which allows us to reformulate the *Z* variable as follows:

$$Z=\mu Z+\sigma_{Z}*\tilde{Z}$$

where $\tilde{Z}$ is the centralized *Z* variable (mean = 0, standard deviation = 1). Using linear approximation, we may obtain:

$$\frac{X}{\mu Z+\sigma_{Z}*\tilde{Z}}\approx \frac{X}{\mu Z}-\frac{1}{\mu Z^{2}}\sigma_{Z}*\tilde{Z}*X$$

Now:

$$\sigma_{S}{}^{2}=E\left({\left(\frac{X}{Z}\right)}^{2}\right)-{\left(E\left(\frac{X}{Z}\right)\right)}^{2}$$

$$\sigma_{S}{}^{2}\approx E\left({\left(\frac{X}{\mu Z}-\frac{1}{\mu Z^{2}}\sigma_{Z}*\tilde{Z}*X\right)}^{2}\right)-{\left(E\left(\frac{X}{Z}\right)\right)}^{2}$$

$$E\left({\left(\frac{X}{\mu Z}-\frac{1}{\mu Z^{2}}\sigma_{Z}*\tilde{Z}*X\right)}^{2}\right)=\frac{1}{\mu Z^{2}}E{\left(X\right)}^{2}-2\frac{dZ}{\mu Z^{2}}E\left(X^{2}\tilde{Z}\right)+\frac{\sigma_{Z}{}^{2}}{\mu Z^{4}}E\left(X^{2}{\tilde{Z}}^{2}\right)=I+II+III$$

$$I.\;\;\;E{\left(X\right)}^{2}=\left(\sigma_{X}{}^{2}+\mu X^{2}\right)$$

Variables are uncorrelated and expected value of $\tilde{Z}$ is equal to 0:

$$II.\;\;E\left(X^{2}\tilde{Z}\right)=0$$

Again, variables are uncorrelated and the standard deviation of $\tilde{Z}$ is equal to 1:

$$III.\;\;E\left(X^{2}{\tilde{Z}}^{2}\right)\approx E\left(X^{2}\right)E\left({\tilde{Z}}^{2}\right)=\left(\sigma_{X}{}^{2}+\mu X^{2}\right)\;$$

Using linearization, we can approximate:

$$E\left(\frac{X}{Z}\right)\approx E\left(\frac{X}{\mu Z}-\frac{1}{\mu Z^{2}}\sigma_{Z}*\tilde{Z}*X\right)=\frac{\mu X}{\mu Z}$$

Finally:

$$\sigma_{S}{}^{2}\approx \left(\sigma_{X}{}^{2}+\mu X^{2}\right)\left(\frac{\sigma_{Z}{}^{2}}{\mu Z^{4}}+\frac{1}{\mu Z^{2}}\right)-\left(\frac{\mu X^{2}}{\mu Z^{2}}\right)$$

**Probability for the confidence interval:**

As demonstrated by Rudzki and Leś, measurements may follow a log-normal distribution [7]. In such a case, the confidence interval can be calculated using logarithmic transformation, which yields a normal distribution of the stability. In order to keep the model simple, from now on we will assume the normal distribution of the stability.

Let us denote the standard deviation in stability as $s_{S}$. Under the assumption o the normal distribution, the 90% confidence interval of stability has the following form:

$$CI=\mu_{S}\pm \frac{s_{S}\;k\;}{\sqrt{n}}$$

where:

*µ_S_* is the mean value of stability and k is the value of the Student t-distribution quantile at a 0.1 significance level for *n*−1 degrees of freedom (*df*). In the presented work, we consider only stable analytes, i.e., *µ_S_* =100. The probability that the confidence interval is in the 85–115% interval:

P(CI ⊂[85; 115])

is equivalent to:

$$P\left(\frac{s_{S}\;k}{\sqrt{n}}<15\right)=P\left(s_{S}{}^{2}<{\left(\frac{15\;\sqrt{n}}{k}\right)}^{2}\right)$$

Assuming that the true standard deviation in stability is $\sigma_{S}$:

$$P\left(s_{S}{}^{2}<{\left(\frac{15\;\sqrt{n}}{k}\right)}^{2}\right)=P(\frac{s_{S}{}^{2}\left(n-1\right)}{\sigma_{S}{}^{2}}<\frac{225\;n\;\left(n-1\right)}{\sigma_{S}{}^{2}k^{2}})$$

where:

$$\frac{s^{2}\left(n-1\right)}{\sigma_{S}{}^{2}}\;\sim \;chi^{2}\left(n-1\right)$$

As a result:

$$P\left(CI\;\subset \left[85;\;115\right]\right)=\chi_{n-1}\left(\frac{225\;n\;\left(n-1\right)}{k^{2}\;\sigma_{S}{}^{2}}\right)$$

where *χ_n_*_−1_ is the cumulative distribution function of the chi-square distribution for df = *n* − 1.
